# Evaluation of NTRK Gene Fusion by Five Different Platforms in Triple-Negative Breast Carcinoma

**DOI:** 10.3389/fmolb.2021.654387

**Published:** 2021-08-19

**Authors:** Shafei Wu, Xiaohua Shi, Xinyu Ren, Kaimi Li, Junyi Pang, Zhiyong Liang

**Affiliations:** Department of Pathology, State Key Laboratory of Complex Severe and Rare Disease, Molecular Pathology Research Center, Peking Union Medical College Hospital, Chinese Academy of Medical Sciences and Peking Union Medical College, Beijing, China

**Keywords:** IHC, NGS, RT-PCR, FISH, NTRK fusion gene, triple-negative breast carcinoma

## Abstract

Triple-negative breast carcinoma (TNBC) is an aggressive disease that has a poor prognosis since it lacks effective treatment methods. Neurotrophic tyrosine receptor kinase (NTRK) fusion genes are excellent candidates for targeted RTK inhibitor therapies and there are available targeted therapy drugs for the treatment of TRK fusion-positive tumors in a tumor agnostic pattern. Our study was designed to investigate the NTRK gene fusion status in TNBC patients and to determine whether RTK-targeted therapies are suitable for TNBC patients. A total of 305 TNBC patients were enrolled in our study. IHC was employed as a prescreening method, and IHC positive cases were further submitted for evaluation by FISH, RT-PCR, and NGS methods. NTRK IHC was evaluated successfully in 287 of the 305 cases, and there were 32 (11.15%) positive cases. FISH was carried out in the 32 IHC positive cases. There were 13 FISH-positive cases if the threshold was set as >15% of the 100 counted tumor cells having a split orange and green signal with more than one signal diameter. There were only 2 FISH-positive cases if the cutoff value was defined as >15% of the counted tumor cells having a split signal with more than two signal diameter widths. One of the FISH-positive cases had a separate NTRK3 FISH signal in 88% of the tumor cells, and its IHC result was strong nuclear staining in all the tumor cells. After evaluation of the morphology, it was re-diagnosed as secretory breast carcinoma, and the NGS result confirmed that it had a NTRK3-ETV6 fusion gene. The other FISH-positive cases were all negative for NTRK gene fusion in the NGS or RT-PCR examination. The NTRK gene fusion rate was low in our TNBC cohort. NTRK gene fusion may be a rare event in TNBC. The high false-positive rate of NTRK gene fusion detected by IHC questions its role as a prescreening method in TNBC. More data may be needed to determine a suitable threshold for NTRK FISH in TNBC in the future. More studies are needed to confirm whether RTK-targeted therapies are appropriate treatments for TNBC patients.

## Introduction

Breast carcinoma is the most common carcinoma in females. It had an incidence of approximately 2.1 million new cases worldwide in 2018 (Ahmad, 2019). Treatment decision is mainly determined by the hormone and HER2 status when a patient presents in an advanced clinical stage. Triple-negative breast carcinoma (TNBC) is a subtype of breast carcinoma with hormone receptor immunohistochemistry (IHC) stains of less than 1% for estrogen receptors (ER) and progesterone receptors (PR) and is devoid of HER2 protein overexpression or *HER2* gene amplification (or both) (Bergin and Loi, 2019). TNBC is a special subgroup of breast carcinoma which has a poor prognosis, early recurrence, and high metastasis rates. TNBC patients usually present with advanced clinical stage, large tumor size, and poor Nottingham prognostic index when evaluated using pathological criteria at diagnosis (Li et al., 2013). Systemic chemotherapy is the mainstay treatment since TNBC lacks effective targeted therapies. TNBC is always a hotspot of investigation owing to the above-mentioned characteristics, and our study focuses on it too.

The neurotrophic tyrosine receptor kinase (NTRK) gene encodes three different tropomyosin receptor kinases, TRKA, TRKB, and TRKC. These proteins play an important role in the physiology of the development and function of the nervous system (Stephen, 2008). The proteins are structured into three components: the extracellular ligand-binding domain, the transmembrane part, and the intracellular kinase domain. The intracellular domain can undergo homodimerization upon ligand binding or gene fusion caused by chromosomal translocation. Aberrant fusion of the NTRK 3′ kinase domain with the other genes can lead to ligand-independent activation of the NTRK gene and constitutively cause an increase in proliferation and decreased apoptosis of the tumor cells (Nakagawara, 2001). Patients with a positive NTRK fusion gene are excellent candidates for targeted RTK inhibitor therapies. To date, two drugs (entrectinib and larotrectinib) have been approved by the FDA for the treatment of TRK fusion-positive tumors in a tumor agnostic pattern. Secretory breast carcinoma patients who have a high incidence of NTRK fusion gene have shown an excellent clinical response to these targeted drugs in clinical trials (Drilon et al., 2018; Scott, 2019). Secretory breast carcinoma is characterized by NTRK3-ETV6 gene fusion (Vasudev and Onuma, 2011). However, the incidence of NTRK gene fusion in other types of breast carcinoma is low, ranging from 0 to 0.08% (Remoué et al., 2019; Vranic et al., 2019; Rosen et al., 2020). But then again, few reports have been focusing on exploring the NTRK fusion rate in TNBC until now.

Tumors harboring NTRK fusion genes can be divided into two groups: one is a special type of tumor that has a high frequency of NTRK fusion genes, including secretory carcinoma of the breast and salivary glands, congenital mesoblastic nephroma, and infant fibrosarcoma (Stransky et al., 2014; Kheder and Hong, 2018). The other is the common tumor in which NTRK fusion is a rare event (Gatalica et al., 2019). There are different methods for the detection of NTRK fusion, such as immunohistochemistry (IHC), fluorescence *in situ* hybridization (FISH), reverse transcription-polymerase chain reaction (RT-PCR), and next-generation sequencing (NGS). The clinical trials did not employ a specific or uniform diagnostic test, and there are no approved companion diagnostic assays. There are recommendations for the methods of identifying NTRK fusion-positive patients in the common tumor types, such as the ESMO and the Japan Society of Clinical Oncology, and the Japanese Society of Medical Oncology (Marchio et al., 2019; Naito et al., 2020). The IHC method can be used as a prescreening method in common tumors with a low incidence of NTRK fusion. Although targeted therapy drugs are histologically agnostic, whether the IHC methods are also histology-based triage needs to be evaluated.

In our study, we evaluated NTRK gene fusion in TNBC patient samples using the NTRK IHC method as a prescreening method. Other methods, including FISH, RT-PCR, and NGS, were carried out in the IHC positive samples to determine the final NTRK fusion status in TNBC.

## Materials and Methods

### Patient Selection

Three hundred and five patients who underwent surgery between January 2011 and December 2014 at Peking Union Medical College Hospital (Beijing, China) were enrolled in our study. All cases had an IHC profile of less than 1% for ER and PR and were devoid of HER2 protein overexpression or *HER2* gene amplification (or both). The average age of the TNBC patients were 49 years old. None of the patients under evaluation had a history of taking NTRK targeted therapy drugs.

This study was approved by the Institutional Review Board of Peking Union Medical College Hospital.

### Tissue Microarray Construction

The selective areas of representative morphology of the hematoxylin-eosin staining slides were labeled. The corresponding formalin-fixed paraffin-embedded (FFPE) primary tumor samples were obtained from the Department of Pathology. A tissue microarray construction machine (Quick-Ray UT-06, UNITMA) was used, and two core-tissue biopsies of 2.0 mm diameter were collected for each sample.

### Immunohistochemistry

NTRK immunohistochemical staining was performed using the antibody clone EPR17341 (Roche, Tusan, United States) to assess NTRK1, NTRK2, and NTRK3 protein expression in the FFPE samples. Positive results were defined as staining above background in at least 1% of tumor cells in any pattern, including membranous, cytoplasmic, perinuclear, or nuclear.

## FISH

Representative FFPE samples were cut into 4-um thick slides, and FISH was performed using the Thermo-Brite Elite automated FISH slide prep system (Leica, Richmond, CA, United States). The FISH break-apart probes used in our study included NTRK1, NTRK2, and NTRK3 Break Apart FISH Probe (ZytoVision GmbH, Bremerhaven, Germany). The results were evaluated using the cytoVision DM6000B fluorescent microscope system (Leica, Biosystem, Buffalo Grove, IL). One hundred tumor nuclei per case were calculated and the percentage of the positive signals was calculated in each case using two different criteria. One was a split of two or more signal widths apart between the orange and green signals in more than 15% of the tumor cells, and the other was a split of more than one signal width apart between the orange and green signals in more than 15% of the tumor cells.

## NGS

DNA was extracted from FFPE tissues using the QIAamp DNA FFPE Tissue Kit (Qiagen) according to the manufacturer’s instructions. After fragmentation with a Covaris S2 ultrasonicator (Covaris, United States) to generate fragments with a 300-bp peak, we performed library construction reactions to generate sequencing libraries using the NEBNext^®^ Ultra^TM^ DNA Library Prep Kit for Illumina^®^ (NEB) according to the manufacturer’s instructions. Then, we enriched the library DNA for targeted regions using customized probe sets (Integrated DNA Technologies, IDT) according to the manufacturer’s instructions. The DNA libraries were then sequenced with a paired-end 2 × 100 bp protocol aiming for an average coverage of 20 × and 100 × for the tumor DNA, respectively. All the final DNA libraries were subsequently sequenced on the Gene + Seq-2000 to generate approximately 6.2 Gb data. MuTect2 (3.4-46-gbc02625) was used to call single nucleotide variants (SNVs), while GATK was employed to call small insertions and deletions (indels). Copy number variations (CNVs) were detected using Contra (2.0.8). Structure variations (SVs) were detected with BreakDancer. All final candidate variants were verified with an integrative genomics viewer browser. After annotation, the variants were cross-referenced with those in the 1000 Genomes Project, GAD, dbSNP, and ExAC.

The DNA-based NGS assay used at Geneplus interrogates whole exon region in NTRK1/2/3, and introns 8–11 in NTRK1, intron 12 in NTRK2, and introns 4–6 in ETV6, the most common NTRK3 fusion partner. However, because of the aforementioned issues involving coverage of the NTRK3 introns, NTRK3 fusion coverage selected the breakpoint region.

For RNA-seq, total RNA was isolated from FFPE using an RNeasy Mini Kit (Qiagen) and RNeasy FFPE Kit (Qiagen), respectively. cDNA synthesis and NGS library preparation were performed using NEBNext^®^ Ultra^TM^ II Directional RNA Library Prep Kit (NEB) following the manufacturer’s protocol, but a substituted adaptor and an index primer were used in Gene + Seq-2000. The library was quantitated using Qubit 3.0 (Life Invitrogen, United States) and quality was assessed using the LabChip GX Touch (PerkinElmer, United States). The libraries were sequenced on the Gene + Seq-2000 with a paired-end 2 × 100 bp protocol resulting in 20 Gb per sample. After removal of terminal adaptor sequences and low-quality data using fastp (version: 0.19.5), and rRNA reads were removed by aligning clean reads to the rRNA database (downloaded from NCBI) by using bowtie2 (version:2.2.8), clean reads without known rRNA were aligned to the reference human genome (hg19) through STAR (version 020201). Fusions were detected using a customized version of Arriba 1.1.0. and annotated using the in-house software annoFilterArriba (version:1.0.0) with the NCBI release 104 database. All final candidate fusions were manually verified with an integrative genomics viewer browser. A series of quality control metrics were computed using RNA-SeQC (Gatalica et al., 2019) assessment. A threshold of ≥ 80 million mapped reads and ≥ 10 million junction reads per sample was set.

## RT-PCR

The NTRK Gene Fusions Detection Kit (AmoyDx) was used in this study. The kit can qualitatively detect 109 fusions in NTRK1, NTRK2, and NTRK3. There were three major steps, including RNA extraction, reverse PCR, and DNA amplification. There are eight NTRK PCR mix tubes that contain fusion detection and internal control systems. The fusion detection system includes primers and FAM-labeled probes specific for NTRK1/2/3 gene fusions. The internal control system contained primers and a VIC-labeled probe for detection of reference genes to reveal the RNA quality and presence of PCR inhibitors that may lead to false-negative results. Reverse transcription and amplification PCR were run on an ABI 7500 PCR machine. For the NTRK PCR mix, FAM Ct values ≤25 were considered positive. Detailed information on the NTRK fusion types examined by the RT-PCR kit is summarized in Table 1.

**TABLE 1:** The detailed information on the NTRK fusion types examined by the RT-PCR kit.

**Tube**	**Detected target**	**Fusion type**
➀	NTRK1 Fusion	TP53 exon8;ins6 NTRK1 exon8
		TP53 exon9;ins6 NTRK1 exon8
		TP53 exon10;ins6 NTRK1 exon8
		TP53 exon11;ins6 NTRK1 exon8
		CTRC exon2;NTRK1 exon8
		IRF2BP2 exon1;NTRK1 exon8
		LRRC71 exon1;NTRK1 exon8
		LMNA exon2;NTRK1 exon11
		LMNA exon3;NTRK1 exon11
		LMNA exon5;NTRK1 exon11
		LMNA exon10;NTRK1 exon11
		LMNA exon11 del150;NTRK1 exon11
		PPL exon21;NTRK1 exon11
		GRIPAP1 exon22;NTRK1 exon11
		BCAN exon13;NTRK1 exon11
➁	NTRK1 Fusion	TFG exon5;NTRK1 exon9
		TPR exon21;NTRK1 exon9
		TFG exon4;NTRK1 exon9
		TPM3 exon10;NTRK1 exon9
		AFAP1 exon4;NTRK1 exon9
		TRIM63 exon8;NTRK1 exon9
		TPM3 exon8;NTRK1 exon10
		SQSTM1 exon2;NTRK1 exon10
		SQSTM1 exon5;NTRK1 exon10
		TPR exon10;NTRK1 exon10
		TPR exon16 del54;NTRK1 ins13 exon10
		TPR exon21;NTRK1 exon10
		CD74 exon8;NTRK1 exon10
		IRF2BP2 exon1;NTRK1 exon10
		IRF2BP2 exon1 del48;NTRK1 exon10
		PPL exon21;NTRK1 exon10
		PEAR1 exon15;NTRK1 exon10
		TFG exon5;NTRK1 exon10
		GRIPAP1 exon22;NTRK1 exon10
		TFG exon6;NTRK1 exon10
		F11R exon4;NTRK1 exon10
		F11 exon4;NTRK1 exon10
		SQSTM1 exon6;NTRK1 exon10
		ARHGEF2 exon21;NTRK1 exon10
		CHTOP exon5;NTRK1 exon10
		NFASC exon21;NTRK1 exon10
		TPM3 exon7 del39;NTRK1 exon10
		BCAN exon12;NTRK1 exon10
		PPL exon11;NTRK1 exon13
➂	NTRK1 Fusion	TPM3 exon8;NTRK1 exon12
		LMNA exon6 del172;NTRK1 exon12
		MPRIP exon21;NTRK1 exon12
		SSBP2 exon12;NTRK1 exon12
		LMNA exon2;NTRK1 exon12
		LMNA exon4;NTRK1 exon12
		LMNA exon8;NTRK1 exon12
		LMNA exon10;NTRK1 exon12
		LMNA exon12;NTRK1 exon12
		MPRIP exon14;NTRK1 exon12
		MPRIP exon18;NTRK1 exon12
		TPR exon6;NTRK1 exon12
		GRIPAP1 exon22;NTRK1 exon12
		SCYL3 exon11;NTRK1 exon12
		MEF2D exon9;NTRK1 exon12
		AMOTL2 exon6;NTRK1 exon12
		PRDX1 exon5;NTRK1 exon12
➃	NTRK1 Fusion	MPRIP exon21;NTRK1 exon14
		LMNA exon2;NTRK1 exon16
➄	NTRK2 Fusion	VCL exon16;NTRK2 exon12
		AFAP1 exon13;NTRK2 exon12
		VCAN exon6; NTRK2 exon12
		NCAA2 exon5;NTRK2 exon13
		NOS1AP exon9; NTRK2 exon13
		TBC1D2 exon6; NTRK2 exon14
➅	NTRK2 Fusion	TRIM24 exon12;NTRK2 exon15
		TRAF2 exon9;NTRK2 exon15
		SQSTM1 exon4;NTRK2 exon15
		ETV6 exon5;NTRK2 exon15
		TLE4 exon7;NTRK2 exon15
		TRIM24 exon12;NTRK2 exon16
		AGBL4 exon6;NTRK2 exon16
		SQSTM1 exon5;NTRK2 exon16
		STRN3 exon7;NTRK2 exon16
		WNK2 exon24;NTRK2 exon16
		QKI exon6;NTRK2 exon16
		STRN exon3;NTRK2 exon16
		GKAP1 exon9; NTRK2 exon16
		KCTD8 exon1;NTRK2 exon16
		PRKAR2A exon2;NTRK2 exon16
		PAN3 exon1;NTRK2 exon17
		SQSTM1 exon5;NTRK2 exon17
		BCR exon1;NTRK2 exon17
➆	NTRK3 Fusion	ETV6 exon4;NTRK3 exon14
		ETV6 exon5;NTRK3 exon14
		EML4 exon2;NTRK3 exon14
		SQSTM1 exon5;NTRK3 exon14
		TFG exon6;NTRK3 exon14
		MYH9 exon31;NTRK3 exon14
		RBPMS exon5;NTRK3 exon14
		BTBD1 exon4; NTRK3 exon14
		SPECC1L exon5;NTRK3 exon14
		VIM exon8;NTRK3 exon14
		STRN exon3;NTRK3 exon14
		STRN3 exon3;NTRK3 exon14
		HNRNPA2B1 exon7;NTRK3 exon14
		AKAP13 exon3;NTRK3 exon14
		ETV6 exon5;NTRK3 exon15
		ETV6 exon4;NTRK3 exon15
		SQSTM1 exon6;NTRK3 exon15
		ETV6 exon6;NTRK3 exon15
➇	NTRK3 Fusion	ETV6 exon4;NTRK3 exon12
		ETV6 exon5;NTRK3 exon13
		ETV6 exon4;NTRK3 exon13
		ETV6 exon5;NTRK3 exon16

## Results

A total of 305 TNBC patients were enrolled in our study. NTRK IHC was evaluated successfully in 287 cases, of which 32 (11.15%) were positive. Six cases showed strong NTRK immunostaining with an average staining percentage of 47% (ranging from 2 to 100%), and the stain was located either in the cytoplasm (5 cases) or nucleus (1 case). There were 15 cases with moderate staining intensity in the tumor cell cytoplasm or nucleus, and the average stain percentage of the tumor cells was 21.67% (ranging from 5 to 40%). The remaining 11 patients had weak cytoplasmic staining in the tumor cells, with an average percentage of 10.27%. Detailed information on IHC staining is summarized in Table 2.

**TABLE 2:** The detail information of the NTRK immunohistochemistry positive cases.

**No**	**Intensity**	**Percentage**	**Location**	**FISH (one diameter)**	**FISH (two diameter)**	**NGS DNA**	**NGS RNA**	**PCR**
1	S	100	Nu	Pos (88%)	Pos (88%)	pos	pos	pos
2	S	80	C	Pos (24%)	Pos (22%)	neg	neg	neg
3	S	60	C	Pos (20%)	Neg	neg	neg	neg
4	S	20	C	Pos (16%)	Neg	neg	neg	neg
5	S	20	C	Neg	Neg	NA	NA	NA
6	S	2	C	Neg	Neg	NA	NA	NA
7	M	40	C	Neg	Neg	NA	NA	NA
8	M	40	C	Neg	Neg	NA	NA	NA
9	M	40	Nu	Neg	Neg	NA	NA	NA
10	M	40	C	Pos (20%)	Neg	neg	NA	NA
11	M	30	C	Pos (22%)	Neg	neg	NA	NA
12	M	30	C	Neg	Neg	NA	NA	NA
13	M	25	C	Neg	Neg	NA	NA	NA
14	M	20	C	Neg	Neg	NA	NA	NA
15	M	10	C	Neg	Neg	NA	NA	NA
16	M	10	C	Neg	Neg	NA	NA	NA
17	M	10	C	Pos (24%)	Neg	neg	neg	neg
18	M	10	C	Pos (26%)	Neg	neg	NA	NA
19	M	10	C	Pos (28%)	Neg	neg	NA	NA
20	M	5	C	Neg	Neg	NA	NA	NA
21	M	5	C	Neg	Neg	NA	NA	NA
22	W	40	C	Neg	Neg	NA	NA	NA
23	W	20	C	Pos (20%)	Neg	neg	NA	NA
24	W	10	C	Neg	Neg	NA	neg	neg
25	W	10	C	Neg	Neg	NA	NA	NA
26	W	5	C	Neg	Neg	NA	NA	NA
27	W	5	C	Neg	Neg	NA	NA	NA
28	W	5	C	Neg	Neg	NA	NA	NA
29	W	5	C	Neg	Neg	NA	NA	NA
30	W	5	C	Pos (18%)	Neg	neg	neg	neg
31	W	5	C	Pos (28%)	Neg	neg	neg	neg
32	W	3	C	Pos (36%)	Neg	neg	NA	NA

*S, strong; M, moderate; W, weak; C, cytoplasm; Nu, nuclear; Neg, negative; Pos, positive; NA, not available. FISH, fluorescence in situ hybridization; NGS, next generation sequencing; PCR, polymease chain reaction*.

**FIGURE 1:**
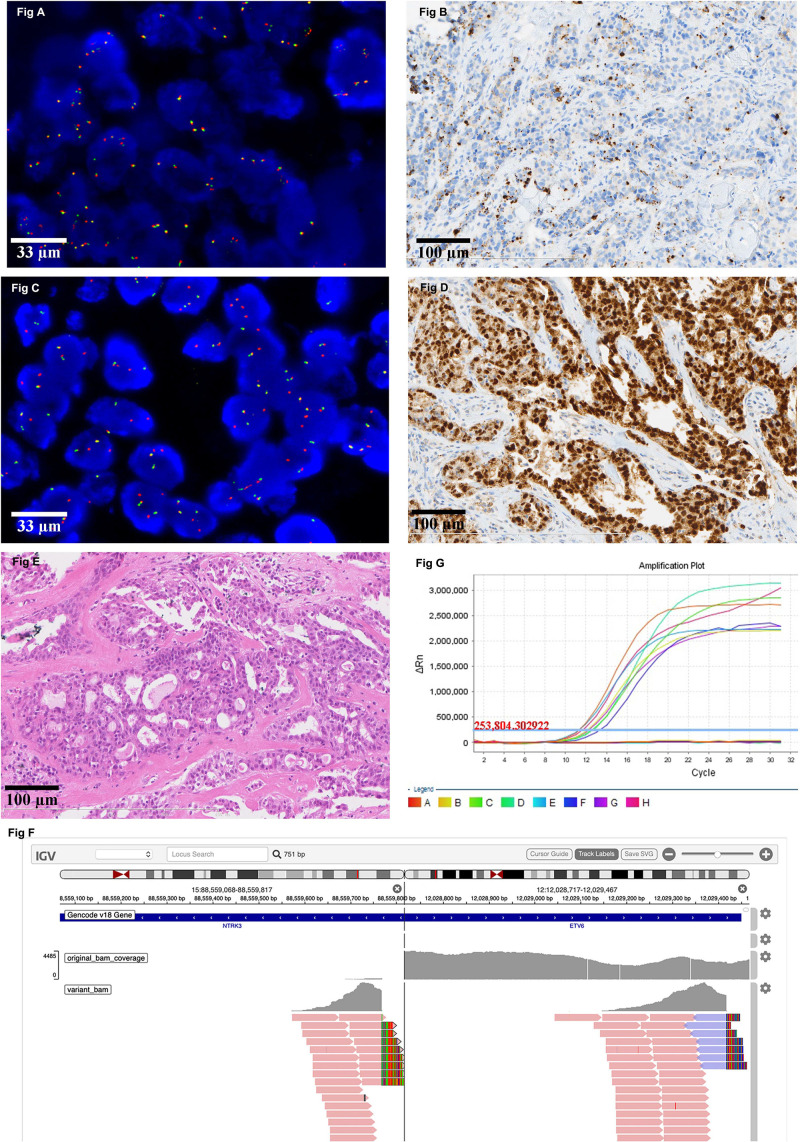
Representative image of NTRK result detected by different platforms. **(A)** There is a positive NTRK1 FISH signal in 22% of the tumor cells (630X). **(B)** The corresponding IHC result of the case in Figure A showed a strong cytoplasmic stain in 80% of the tumor cells (200X). **(C)** There is a positive NTRK3 FISH signal in 88% of the tumor cells (630X). **(D)** The corresponding IHC result of the case in Figure C indicated a strong and diffuse nuclear stain in almost all tumor cells (200X). **(E)** The morphology of the case which the diagnosis was modified from breast carcinoma, no special type, to secretory breast carcinoma (100X). **(F)** Positive NTRK3 result detected by DNA-based NGS. **(G)** Representative image of negative NTRK result detected by RT-PCR.

All IHC positive cases were subjected to FISH testing. If the evaluation threshold was set to be >15% of the tumor cells with a separation width of more than one signal diameter, there were 13(4.5%) positive cases with an average ratio of 28.46%. The highest proportion of the separated signals was 88%, followed by 36% and 28%. If the evaluation threshold was set to be > 15% of the tumor cells with a separation width of more than two signal diameters, there were 2 (0.70%) positive cases with an average ratio of 55%. One of the FISH positive cases had a positive NTRK1 separation signal in 22% of the tumor cells (Figure 1A), while its IHC result showed a strong cytoplasmic stain in 80% of the tumor cells (Figure 1B). The other FISH positive case identified an NTRK3 separation signal in 88% of the tumor cells (Figure 1C), and IHC results indicated a strong and diffuse nuclear stain in almost all tumor cells (Figure 1D). After further evaluation of the HE slides, the latter case was modified from breast carcinoma, no special type to secretory breast carcinoma (Figure 1E).

DNA-based NGS was carried out in 13 cases in which the IHC was positive and FISH could identify separated orange and green signals of more than one diameter width in over 15% of the tumor cells. Besides the one which is confirmed to be secretory breast carcinoma (Figure 1F), the rest of them are all negative for NTRK rearrangement. RNA-based NGS was further evaluated in 7 cases that were negative for NTRK fusion examined by DNA-based NGS and had a good quality of RNA for further analysis. The final results were also negative for NTRK gene rearrangement at the RNA level.

RT-PCR was also carried out in the 7 cases that had been evaluated by RNA-based NGS, and the results were also negative for NTRK rearrangement (Figure 1G).

## Discussion

In our study, we used five different platforms to evaluate NTRK gene rearrangement in TNBC. IHC was used as a pre-screening method with a high false-positive result. DNA-based NGS, RNA-based NGS, and RT-PCR methods did not identify positive NTRK gene rearrangements except in one secretory breast carcinoma case with NTRK3 gene fusion, which was misdiagnosed as TBNC. Different FISH evaluation criteria yield diverse positive results for NTRK gene fusion. Only the NTRK FISH result with a high proportion of split signals in the tumor cells can achieve positive NGS or RT-PCR results.

NTRK gene fusion is reported to be a rare event in common cancer types, such as pancreatic carcinoma and lung cancer, with a reported incidence below 1% (Rosen et al., 2020). There are several massive sequencing results showing that NTRK gene fusion in breast carcinoma ranges from 0 to 0.34%, most of which are focused on breast carcinoma (NOS). As we know, TNBC is a special type of breast carcinoma that lacks effective treatment methods, and at diagnosis, is always at an advanced clinical stage, with high recurrence and metastasis rates. Therefore, exploring new targeted therapy methods for this type of tumor is meaningful. Unfortunately, our preliminary results show that the NTRK fusion rate in TNBC is low. To our knowledge, there is only one report focusing on investigating NTRK gene fusion in TNBC, and its conclusion is similar to ours (Remoué et al., 2019).

Secretory breast carcinomas are a rare type of breast carcinoma, accounting for less than 0.15% of the invasive breast cancers (Lee et al., 2014; Del Castillo et al., 2015). They are characterized by NTRK3-ETV6 gene rearrangement (Vasudev and Onuma, 2011). Secretory breast carcinoma usually presents as a phenotype of triple-negative breast carcinoma with typical features of intracellular and extracellular eosinophilic secretion material. One secretory breast carcinoma was wrongly included in TNBC because of its negative expression of ER, PR, and HER-2 in our study. NTRK IHC showed strong nuclear staining in all the tumor cells and positive NTRK FISH signals in 88% of the tumor cells. NGS also identified NTRK3 fusion signals in this peculiar case. Correct diagnosis of secretory breast carcinoma is important because it is morphologically and immunohistologically different from TNBC. Second, it has targeted therapy for advanced-stage patients because of the high prevalence of characteristic NTRK3-ETV6 gene fusion.

There are different methods to detect the fusion status of NTRK genes, including NGS, RT-PCR, FISH, and IHC (Solomon and Hechtman, 2019). Each one has its own advantages and disadvantages. NGS methods, which include DNA-based NGS and RNA-based NGS, are a massive sequencing method that can tell the corresponding fusion partners. Their disadvantage is the long turnaround time and requirement of a high amount of DNA input and good quality of RNA. Although RT-PCR has a short turnaround time compared to the NGS method, it can only detect the known fusion types of NTRK, and there are currently no commercial testing kits available on the market. The FISH method is limited by the experience of the pathologists and may produce false-negative results if the breakpoints involve non-canonical sites. The IHC method is a labor and time saving method that can be carried out in most pathology labs. However, the absence of a standardized scoring method limits its application in clinical practice. Since there are no uniform examination methods in clinical trials, there is no consensus method for the detection of NTRK gene fusion.

Recently, the ESMO working group proposed a two-step method to detect NTRK fusions in daily practice, and IHC can be used as a pre-screening method in common cancers that have a low prevalence of NTRK fusion (Marchio et al., 2019). In our study, we used the IHC method to screen for NTRK fusion patients in TNBC. The final results showed that the fusion rate of TNBC in our cohort was 11.15%, which is high above the reported level. Further NGS analysis showed that the IHC positive cases were all negative for NTRK rearrangement at the DNA and RNA levels, except for one secretory breast carcinoma case. Our preliminary results question the role of IHC as a prescreening method in TNBC because of its high false positivity rate. This phenomenon was not observed in lung cancer, gallbladder carcinoma, or pancreatic cancer in our parallel study (data not published). Several studies have investigated the sensitivity and specificity of the pan-TRK IHC method vs. the FISH or NGS method, and the results showed that the positive predictive value and negative predictive value are high between the various methods in infant fibrosarcoma, lipofibromatosis-like neural tumor, colorectal cancer, and lung adenocarcinoma (Hechtman et al., 2017; Rudzinski et al., 2018). In Solomen’s report, NTRK IHC specificity was 100% for carcinomas of the colon, lung, thyroid, pancreas, and biliary tract, while decreased specificity was seen in breast and salivary gland carcinomas (82 and 52%, respectively) (Solomon et al., 2019). Some research result indicated that the pan-TRK antibody can predict the NTRK fusion partners according to the staining patterns. For example the ETV6-NTRK3 positive cases were prone to have nuclear staining, like the secretory carcinoma in our study which showed diffuse and strong nuclear stain. While LMNA-NTRK1 fusion positive samples displayed perinuclear expression pattern and TPM-NTRK1 fusions showed membranous staining (Gatalica et al., 2019).

Together with our study results, we found that although the NTRK treatment relies on the specific molecular alteration instead of the histological classification, the prescreening IHC method should be histology-triaged, and IHC is not a suitable prescreening method in TNBC tumor type.

FISH is regarded as the gold standard method for the detection of gene fusion because it can visualize the separation or fusion signals under a microscope, even in poorly preserved FFPE samples. There are currently no consensus criteria for the evaluation of NTRK FISH results. Different criteria have been applied in research papers, for example, a threshold of > 15% of tumor nuclei with a positive signal (i.e., a split signal of a single 3′ orange signal, or a split pattern with 3′ and 5′ signals separated by a distance superior to the diameter of the largest signal) within 100 tumor nuclei (George et al., 2018; Remoué et al., 2019), or cases can be considered positive for gene fusion if >10% or >15% of nuclei display “split-apart signals” (red and green signals should be separated by a distance greater than the size of two hybridization probe signals) (Del Castillo et al., 2015; Marchio et al., 2019). In our study, if the split signal proportion was below 50% of the tumor cells, regardless of the separation signal width (more than one signal diameter or two signal diameters), the NGS or RT-PCR method could not identify NTRK gene fusion in the tumor cells. Only cases with a high proportion of NTRK gene split signals in the FISH examination had positive NGS and RT-PCR results. There have been few reports describing the detailed FISH positive signal proportion in context. One study showed that an average of 55, 75, 70, and 55% of the tumor cells were positive in the FISH examination within the known fusion-positive cases tested by NGS (Kirchner et al., 2020). Taken together, the FISH positive threshold of the NTRK gene needs to be further evaluated to reach a suitable cutoff value.

The retrospective nature of our study is one of its limitations. We lacked information on the available targeted drugs. The specimens included in our study were at least 5-year-old archived FFPE samples, which were not suitable for NGS analysis because of the limitation of DNA and RNA quality. Finally, since not all the cases underwent NGS analysis, there was no accurate false-negative predictive value in our study.

In conclusion, NTRK gene fusion may be a rare event in TNBC. The high false-positive rate of NTRK gene fusion detected by IHC questions its role as a prescreening method in TNBC. More data may be needed to determine a suitable threshold for NTRK FISH in TNBC in the future. More studies are needed to confirm whether RTK-targeted therapies are appropriate treatments for TNBC patients.

## Data Availability Statement

The authors declare that the datasets presented in this article are not readily available because of the restriction of uploading human genetic resources. Requests to access the datasets should be directed to the corresponding author ZL, liangzhiyong1220@yahoo.com.

## Ethics Statement

The studies involving human participants were reviewed and approved by the Medical Ethical Committee of the Peking Union Medical College Hospital. Written informed consent for participation was not required for this study in accordance with the national legislation and the institutional requirements.

## Author Contributions

SW and XS performed experiments and data analysis and wrote manuscript. XR and KL performed experiments and data analysis. JP performed IHC staining. ZL conceptualized the study design and manuscript writing. All authors contributed to the article and approved the submitted version.

## Conflict of Interest

The authors declare that the research was conducted in the absence of any commercial or financial relationships that could be construed as a potential conflict of interest.

## Publisher’s Note

All claims expressed in this article are solely those of the authors and do not necessarily represent those of their affiliated organizations, or those of the publisher, the editors and the reviewers. Any product that may be evaluated in this article, or claim that may be made by its manufacturer, is not guaranteed or endorsed by the publisher.
